# 2-[1-(9-Anthrylmeth­yl)-1*H*-pyrazol-3-yl]pyridine

**DOI:** 10.1107/S1600536809039427

**Published:** 2009-10-03

**Authors:** Shi-Lu Zhang, Bo-Yan Xie, Da-Bin Qin

**Affiliations:** a School of Chemistry and Chemical Engineering, China West Normal University, Nanchong 637002, People’s Republic of China

## Abstract

The title compound, C_23_H_17_N_3_, can be used in coordination chemistry. The anthracene ring makes dihedral angles of 86.08 (5) and 76.63 (6)°, respectively, with the pyridine and pyrazole rings. The dihedral angle between the pyrazole and pyrimidine rings is 11.79 (7)°. In the structure, weak inter­molecular C—H⋯N hydrogen bonds are observed.

## Related literature

For the synthesis, see: Amoroso *et al.* (1994 ▶); Amir *et al.* (2008 ▶); Stell (2005 ▶); Ward *et al.* (2001 ▶). For related structures, see: Liu *et al.* (2008 ▶).
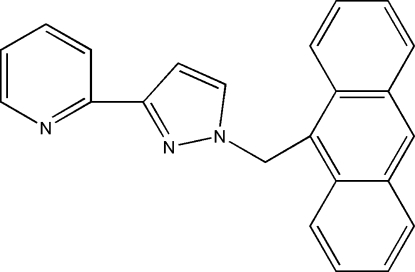


## Experimental

### 

#### Crystal data

C_23_H_17_N_3_
*M* *_r_* = 335.40
Monoclinic, 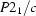

*a* = 13.736 (3) Å
*b* = 13.679 (3) Å
*c* = 8.913 (2) Å
β = 98.496 (3)°
*V* = 1656.2 (7) Å^3^
*Z* = 4
Mo *K*α radiation
μ = 0.08 mm^−1^
*T* = 93 K
0.40 × 0.33 × 0.20 mm

#### Data collection

Rigaku SPIDER diffractometer
Absorption correction: none
13094 measured reflections
3777 independent reflections
3156 reflections with *I* > 2σ(*I*)
*R*_int_ = 0.034

#### Refinement

*R*[*F* ^2^ > 2σ(*F* ^2^)] = 0.049
*wR*(*F* ^2^) = 0.122
*S* = 1.00
3777 reflections
235 parameters
H-atom parameters constrained
Δρ_max_ = 0.23 e Å^−3^
Δρ_min_ = −0.22 e Å^−3^

### 

Data collection: *RAPID-AUTO* (Rigaku/MSC, 2004 ▶); cell refinement: *RAPID-AUTO*; data reduction: *RAPID-AUTO*; program(s) used to solve structure: *SHELXS97* (Sheldrick, 2008 ▶); program(s) used to refine structure: *SHELXL97* (Sheldrick, 2008 ▶); molecular graphics: *ORTEP-3 for Windows* (Farrugia, 1997 ▶); software used to prepare material for publication: *CrystalStructure* (Rigaku/MSC, 2004 ▶).

## Supplementary Material

Crystal structure: contains datablocks global, I. DOI: 10.1107/S1600536809039427/at2873sup1.cif

Structure factors: contains datablocks I. DOI: 10.1107/S1600536809039427/at2873Isup2.hkl

Additional supplementary materials:  crystallographic information; 3D view; checkCIF report

## Figures and Tables

**Table 1:** Hydrogen-bond geometry (Å, °)

*D*—H⋯*A*	*D*—H	H⋯*A*	*D*⋯*A*	*D*—H⋯*A*
C5—H5⋯N2^i^	0.95	2.55	3.312 (2)	138

Symmetry code: (i) 
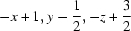
.

**Fig. 1.**
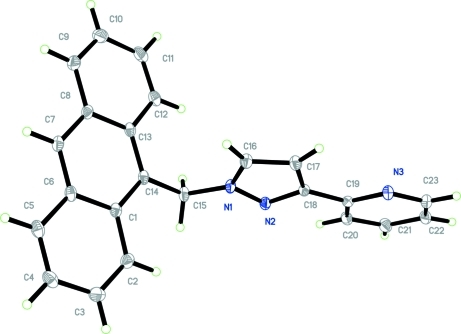
The molecular structure of the title compound, showing 30% probability displacement ellipsoids and the atomic numbering.

## supplementary crystallographic information

### Comment

In recent years, scientists have paid much attention to the synthetic approach and the structural control of coordination architectures with ligands based on pyrazolyl-pyridine chelating units. (Stell, 2005; Ward *et al.*, 2001). In addition, some pyrazole-derived ligands are useful in medication. (Amir *et al.*, 2008). We report herein the synthesis and crystal structure of the title compound (I). Bond lengths and angles in (I) (Fig. 1) are normal.

The dihedral angles formed by the anthracene ring between pyridine and the pyrazole rings are 86.08 (5)° and 76.63 (6)°, respectively. Pyrazole makes a dihedral angle of 11.79 (7)° with pyridine ring .

Weak intermolecular C—H···N hydrogen bonds between molecules are observed.

### Experimental

The title compound was prepared according to the reported procedure of Amoroso *et al.* (1994). Yellow single crystals suitable for X-ray diffraction were obtained by recrystallization from dichloromethane and pPetroleum ether.

### Refinement

H atoms were placed in calculated positions with C—H = 0.95–0.9900 Å, and refined in riding mode with *U*_iso_(H) = 1.2*U*_eq_(C).

### Crystal data

**Table:**

C_23_H_17_N_3_	*F*(000) = 704
*M**_r_* = 335.40	*D*_x_ = 1.345 Mg m^−^^3^
Monoclinic, *P*2_1_/*c*	Mo *K*α radiation, λ = 0.71073 Å
Hall symbol: -P 2ybc	Cell parameters from 4717 reflections
*a* = 13.736 (3) Å	θ = 3.3–27.5°
*b* = 13.679 (3) Å	µ = 0.08 mm^−^^1^
*c* = 8.913 (2) Å	*T* = 93 K
β = 98.496 (3)°	Prism, yellow
*V* = 1656.2 (7) Å^3^	0.40 × 0.33 × 0.20 mm
*Z* = 4	

### Data collection

**Table:**

Rigaku SPIDER diffractometer	3156 reflections with *I* > 2σ(*I*)
Radiation source: Rotating Anode	*R*_int_ = 0.034
graphite	θ_max_ = 27.5°, θ_min_ = 3.3°
ω scans	*h* = −17→17
13094 measured reflections	*k* = −17→15
3777 independent reflections	*l* = −11→11

### Refinement

**Table:**

Refinement on *F*^2^	Primary atom site location: structure-invariant direct methods
Least-squares matrix: full	Secondary atom site location: difference Fourier map
*R*[*F*^2^ > 2σ(*F*^2^)] = 0.049	Hydrogen site location: inferred from neighbouring sites
*wR*(*F*^2^) = 0.122	H-atom parameters constrained
*S* = 1.00	*w* = 1/[σ^2^(*F*_o_^2^) + (0.0596*P*)^2^ + 0.333*P*] where *P* = (*F*_o_^2^ + 2*F*_c_^2^)/3
3777 reflections	(Δ/σ)_max_ = 0.001
235 parameters	Δρ_max_ = 0.23 e Å^−^^3^
0 restraints	Δρ_min_ = −0.22 e Å^−^^3^

### Special details

**Table:**

Geometry. All e.s.d.'s (except the e.s.d. in the dihedral angle between two l.s. planes) are estimated using the full covariance matrix. The cell e.s.d.'s are taken into account individually in the estimation of e.s.d.'s in distances, angles and torsion angles; correlations between e.s.d.'s in cell parameters are only used when they are defined by crystal symmetry. An approximate (isotropic) treatment of cell e.s.d.'s is used for estimating e.s.d.'s involving l.s. planes.
Refinement. Refinement of *F*^2^ against ALL reflections. The weighted *R*-factor *wR* and goodness of fit *S* are based on *F*^2^, conventional *R*-factors *R* are based on *F*, with *F* set to zero for negative *F*^2^. The threshold expression of *F*^2^ > σ(*F*^2^) is used only for calculating *R*-factors(gt) *etc*. and is not relevant to the choice of reflections for refinement. *R*-factors based on *F*^2^ are statistically about twice as large as those based on *F*, and *R*- factors based on ALL data will be even larger.

### Fractional atomic coordinates and isotropic or equivalent isotropic displacement parameters (Å^2^)

**Table:**

	*x*	*y*	*z*	*U*_iso_*/*U*_eq_	
N1	0.25794 (8)	0.55924 (8)	0.72942 (13)	0.0216 (3)	
N2	0.22498 (8)	0.64246 (8)	0.78636 (13)	0.0219 (3)	
N3	0.02717 (8)	0.65643 (9)	1.01184 (13)	0.0264 (3)	
C1	0.46616 (10)	0.43366 (9)	0.66749 (15)	0.0216 (3)	
C2	0.51916 (10)	0.47480 (11)	0.80358 (16)	0.0280 (3)	
H2	0.4936	0.5309	0.8469	0.034*	
C3	0.60559 (11)	0.43505 (11)	0.87224 (17)	0.0313 (4)	
H3	0.6392	0.4640	0.9621	0.038*	
C4	0.64588 (11)	0.35107 (11)	0.81111 (17)	0.0308 (3)	
H4	0.7064	0.3245	0.8595	0.037*	
C5	0.59810 (10)	0.30887 (11)	0.68378 (16)	0.0270 (3)	
H5	0.6256	0.2526	0.6437	0.032*	
C6	0.50709 (10)	0.34731 (10)	0.60844 (15)	0.0229 (3)	
C7	0.45791 (10)	0.30295 (10)	0.47849 (16)	0.0241 (3)	
H7	0.4848	0.2453	0.4414	0.029*	
C8	0.37068 (10)	0.34050 (10)	0.40140 (15)	0.0235 (3)	
C9	0.32166 (11)	0.29600 (11)	0.26587 (17)	0.0311 (3)	
H9	0.3473	0.2373	0.2304	0.037*	
C10	0.23940 (12)	0.33579 (13)	0.18707 (18)	0.0375 (4)	
H10	0.2084	0.3053	0.0967	0.045*	
C11	0.19940 (11)	0.42289 (12)	0.23918 (17)	0.0341 (4)	
H11	0.1421	0.4510	0.1828	0.041*	
C12	0.24260 (10)	0.46640 (11)	0.36920 (16)	0.0282 (3)	
H12	0.2138	0.5238	0.4034	0.034*	
C13	0.33022 (10)	0.42817 (10)	0.45598 (15)	0.0221 (3)	
C14	0.37813 (10)	0.47367 (10)	0.58909 (15)	0.0219 (3)	
C15	0.33882 (10)	0.56910 (10)	0.63969 (17)	0.0255 (3)	
H15A	0.3934	0.6050	0.7008	0.031*	
H15B	0.3157	0.6090	0.5488	0.031*	
C16	0.20496 (10)	0.48050 (10)	0.76085 (16)	0.0247 (3)	
H16	0.2146	0.4149	0.7314	0.030*	
C17	0.13437 (10)	0.51320 (10)	0.84353 (16)	0.0251 (3)	
H17	0.0859	0.4755	0.8832	0.030*	
C18	0.14980 (9)	0.61436 (10)	0.85633 (14)	0.0209 (3)	
C19	0.09372 (9)	0.68884 (10)	0.92693 (15)	0.0217 (3)	
C20	0.10796 (10)	0.78813 (11)	0.90066 (17)	0.0282 (3)	
H20	0.1565	0.8087	0.8419	0.034*	
C21	0.05025 (11)	0.85594 (11)	0.96156 (18)	0.0322 (4)	
H21	0.0578	0.9238	0.9439	0.039*	
C22	−0.01841 (10)	0.82348 (11)	1.04831 (17)	0.0299 (3)	
H22	−0.0592	0.8684	1.0914	0.036*	
C23	−0.02639 (10)	0.72459 (11)	1.07094 (17)	0.0289 (3)	
H23	−0.0730	0.7030	1.1326	0.035*	

### Atomic displacement parameters (Å^2^)

**Table:**

	*U*^11^	*U*^22^	*U*^33^	*U*^12^	*U*^13^	*U*^23^
N1	0.0219 (6)	0.0206 (6)	0.0242 (6)	0.0015 (4)	0.0092 (5)	−0.0014 (5)
N2	0.0204 (6)	0.0214 (6)	0.0252 (6)	0.0009 (4)	0.0069 (5)	−0.0031 (4)
N3	0.0232 (6)	0.0312 (7)	0.0264 (6)	0.0013 (5)	0.0092 (5)	−0.0025 (5)
C1	0.0219 (7)	0.0223 (7)	0.0229 (7)	−0.0025 (5)	0.0104 (5)	0.0026 (5)
C2	0.0295 (8)	0.0317 (8)	0.0247 (7)	−0.0045 (6)	0.0107 (6)	−0.0008 (6)
C3	0.0284 (7)	0.0422 (9)	0.0239 (7)	−0.0092 (6)	0.0056 (6)	0.0022 (6)
C4	0.0225 (7)	0.0422 (9)	0.0285 (8)	0.0003 (6)	0.0066 (6)	0.0119 (6)
C5	0.0240 (7)	0.0290 (8)	0.0299 (8)	0.0032 (6)	0.0106 (6)	0.0086 (6)
C6	0.0226 (7)	0.0238 (7)	0.0248 (7)	0.0013 (5)	0.0117 (6)	0.0055 (5)
C7	0.0255 (7)	0.0208 (7)	0.0285 (7)	0.0009 (5)	0.0122 (6)	−0.0005 (5)
C8	0.0238 (7)	0.0246 (7)	0.0246 (7)	−0.0030 (5)	0.0114 (6)	−0.0013 (6)
C9	0.0298 (8)	0.0344 (8)	0.0314 (8)	−0.0049 (6)	0.0121 (6)	−0.0075 (6)
C10	0.0301 (8)	0.0536 (11)	0.0292 (8)	−0.0096 (7)	0.0059 (7)	−0.0061 (7)
C11	0.0218 (7)	0.0521 (10)	0.0289 (8)	−0.0026 (7)	0.0052 (6)	0.0086 (7)
C12	0.0223 (7)	0.0332 (8)	0.0308 (8)	0.0006 (6)	0.0094 (6)	0.0065 (6)
C13	0.0207 (6)	0.0237 (7)	0.0241 (7)	−0.0012 (5)	0.0105 (5)	0.0034 (5)
C14	0.0219 (6)	0.0212 (7)	0.0250 (7)	0.0000 (5)	0.0114 (5)	0.0019 (5)
C15	0.0254 (7)	0.0234 (7)	0.0312 (7)	0.0010 (5)	0.0153 (6)	0.0006 (6)
C16	0.0270 (7)	0.0203 (7)	0.0286 (7)	−0.0011 (5)	0.0100 (6)	−0.0002 (6)
C17	0.0238 (7)	0.0253 (7)	0.0279 (7)	−0.0007 (5)	0.0094 (6)	0.0015 (6)
C18	0.0194 (6)	0.0250 (7)	0.0185 (6)	0.0008 (5)	0.0035 (5)	−0.0004 (5)
C19	0.0179 (6)	0.0266 (7)	0.0205 (6)	0.0010 (5)	0.0021 (5)	−0.0033 (5)
C20	0.0226 (7)	0.0282 (8)	0.0350 (8)	−0.0020 (6)	0.0082 (6)	−0.0058 (6)
C21	0.0279 (8)	0.0271 (8)	0.0429 (9)	−0.0007 (6)	0.0092 (7)	−0.0080 (6)
C22	0.0224 (7)	0.0318 (8)	0.0362 (8)	0.0033 (6)	0.0072 (6)	−0.0096 (7)
C23	0.0233 (7)	0.0368 (9)	0.0281 (7)	0.0014 (6)	0.0090 (6)	−0.0061 (6)

### Geometric parameters (Å, °)

**Table:**

N1—N2	1.3513 (15)	C10—C11	1.419 (2)
N1—C16	1.3522 (17)	C10—H10	0.9500
N1—C15	1.4678 (17)	C11—C12	1.359 (2)
N2—C18	1.3391 (17)	C11—H11	0.9500
N3—C23	1.3425 (18)	C12—C13	1.4301 (19)
N3—C19	1.3451 (17)	C12—H12	0.9500
C1—C14	1.4144 (19)	C13—C14	1.4132 (19)
C1—C2	1.4342 (19)	C14—C15	1.5069 (19)
C1—C6	1.4407 (19)	C15—H15A	0.9900
C2—C3	1.366 (2)	C15—H15B	0.9900
C2—H2	0.9500	C16—C17	1.3764 (18)
C3—C4	1.418 (2)	C16—H16	0.9500
C3—H3	0.9500	C17—C18	1.402 (2)
C4—C5	1.353 (2)	C17—H17	0.9500
C4—H4	0.9500	C18—C19	1.4733 (18)
C5—C6	1.4290 (19)	C19—C20	1.397 (2)
C5—H5	0.9500	C20—C21	1.382 (2)
C6—C7	1.391 (2)	C20—H20	0.9500
C7—C8	1.389 (2)	C21—C22	1.378 (2)
C7—H7	0.9500	C21—H21	0.9500
C8—C9	1.429 (2)	C22—C23	1.374 (2)
C8—C13	1.4365 (19)	C22—H22	0.9500
C9—C10	1.353 (2)	C23—H23	0.9500
C9—H9	0.9500		
			
N2—N1—C16	111.88 (11)	C11—C12—H12	119.1
N2—N1—C15	116.73 (11)	C13—C12—H12	119.1
C16—N1—C15	131.29 (11)	C14—C13—C12	122.90 (13)
C18—N2—N1	104.91 (11)	C14—C13—C8	119.77 (12)
C23—N3—C19	116.69 (13)	C12—C13—C8	117.32 (13)
C14—C1—C2	123.78 (13)	C13—C14—C1	120.16 (12)
C14—C1—C6	119.21 (12)	C13—C14—C15	119.33 (12)
C2—C1—C6	117.00 (12)	C1—C14—C15	120.35 (12)
C3—C2—C1	121.45 (14)	N1—C15—C14	114.65 (11)
C3—C2—H2	119.3	N1—C15—H15A	108.6
C1—C2—H2	119.3	C14—C15—H15A	108.6
C2—C3—C4	120.87 (14)	N1—C15—H15B	108.6
C2—C3—H3	119.6	C14—C15—H15B	108.6
C4—C3—H3	119.6	H15A—C15—H15B	107.6
C5—C4—C3	119.94 (14)	N1—C16—C17	107.18 (12)
C5—C4—H4	120.0	N1—C16—H16	126.4
C3—C4—H4	120.0	C17—C16—H16	126.4
C4—C5—C6	121.28 (14)	C16—C17—C18	104.73 (12)
C4—C5—H5	119.4	C16—C17—H17	127.6
C6—C5—H5	119.4	C18—C17—H17	127.6
C7—C6—C5	120.94 (13)	N2—C18—C17	111.30 (11)
C7—C6—C1	119.61 (12)	N2—C18—C19	119.23 (12)
C5—C6—C1	119.44 (13)	C17—C18—C19	129.41 (12)
C8—C7—C6	121.87 (13)	N3—C19—C20	122.57 (12)
C8—C7—H7	119.1	N3—C19—C18	117.01 (12)
C6—C7—H7	119.1	C20—C19—C18	120.38 (12)
C7—C8—C9	121.67 (13)	C21—C20—C19	118.95 (13)
C7—C8—C13	119.34 (13)	C21—C20—H20	120.5
C9—C8—C13	118.95 (13)	C19—C20—H20	120.5
C10—C9—C8	121.36 (14)	C22—C21—C20	118.93 (14)
C10—C9—H9	119.3	C22—C21—H21	120.5
C8—C9—H9	119.3	C20—C21—H21	120.5
C9—C10—C11	120.12 (14)	C23—C22—C21	118.39 (13)
C9—C10—H10	119.9	C23—C22—H22	120.8
C11—C10—H10	119.9	C21—C22—H22	120.8
C12—C11—C10	120.38 (14)	N3—C23—C22	124.45 (14)
C12—C11—H11	119.8	N3—C23—H23	117.8
C10—C11—H11	119.8	C22—C23—H23	117.8
C11—C12—C13	121.85 (14)		
			
C16—N1—N2—C18	0.52 (15)	C12—C13—C14—C15	−2.84 (19)
C15—N1—N2—C18	177.37 (11)	C8—C13—C14—C15	176.04 (11)
C14—C1—C2—C3	−177.87 (13)	C2—C1—C14—C13	−179.68 (12)
C6—C1—C2—C3	1.33 (19)	C6—C1—C14—C13	1.14 (19)
C1—C2—C3—C4	−0.2 (2)	C2—C1—C14—C15	5.00 (19)
C2—C3—C4—C5	−0.6 (2)	C6—C1—C14—C15	−174.18 (11)
C3—C4—C5—C6	0.1 (2)	N2—N1—C15—C14	175.47 (11)
C4—C5—C6—C7	−179.35 (13)	C16—N1—C15—C14	−8.4 (2)
C4—C5—C6—C1	1.1 (2)	C13—C14—C15—N1	83.96 (15)
C14—C1—C6—C7	−2.08 (19)	C1—C14—C15—N1	−100.67 (15)
C2—C1—C6—C7	178.69 (12)	N2—N1—C16—C17	−0.56 (16)
C14—C1—C6—C5	177.49 (11)	C15—N1—C16—C17	−176.82 (13)
C2—C1—C6—C5	−1.75 (18)	N1—C16—C17—C18	0.35 (15)
C5—C6—C7—C8	−178.37 (12)	N1—N2—C18—C17	−0.28 (15)
C1—C6—C7—C8	1.2 (2)	N1—N2—C18—C19	−177.62 (11)
C6—C7—C8—C9	178.47 (12)	C16—C17—C18—N2	−0.04 (15)
C6—C7—C8—C13	0.6 (2)	C16—C17—C18—C19	176.95 (13)
C7—C8—C9—C10	−176.59 (14)	C23—N3—C19—C20	0.30 (19)
C13—C8—C9—C10	1.3 (2)	C23—N3—C19—C18	−177.46 (12)
C8—C9—C10—C11	−0.6 (2)	N2—C18—C19—N3	−171.91 (12)
C9—C10—C11—C12	−0.7 (2)	C17—C18—C19—N3	11.3 (2)
C10—C11—C12—C13	1.5 (2)	N2—C18—C19—C20	10.27 (19)
C11—C12—C13—C14	178.08 (13)	C17—C18—C19—C20	−166.52 (14)
C11—C12—C13—C8	−0.8 (2)	N3—C19—C20—C21	−1.3 (2)
C7—C8—C13—C14	−1.57 (19)	C18—C19—C20—C21	176.38 (13)
C9—C8—C13—C14	−179.47 (12)	C19—C20—C21—C22	1.0 (2)
C7—C8—C13—C12	177.37 (12)	C20—C21—C22—C23	0.2 (2)
C9—C8—C13—C12	−0.53 (18)	C19—N3—C23—C22	1.0 (2)
C12—C13—C14—C1	−178.22 (12)	C21—C22—C23—N3	−1.3 (2)
C8—C13—C14—C1	0.66 (19)		

### Hydrogen-bond geometry (Å, °)

**Table:**

*D*—H···*A*	*D*—H	H···*A*	*D*···*A*	*D*—H···*A*
C5—H5···N2^i^	0.95	2.55	3.312 (2)	138

Symmetry codes: (i) −*x*+1, *y*−1/2, −*z*+3/2.
